# Optimized tumour infiltrating lymphocyte assessment for triple negative breast cancer prognostics

**DOI:** 10.1016/j.breast.2021.02.007

**Published:** 2021-02-17

**Authors:** Maschenka CA. Balkenhol, Francesco Ciompi, Żaneta Świderska-Chadaj, Rob van de Loo, Milad Intezar, Irene Otte-Höller, Daan Geijs, Johannes Lotz, Nick Weiss, Thomas de Bel, Geert Litjens, Peter Bult, Jeroen AWM. van der Laak

**Affiliations:** a Radboud University Medical Center, Radboud Institute for Health Sciences, Department of Pathology, Nijmegen, the Netherlands; b Fraunhofer Institute for Image Computing MEVIS, Lübeck, Germany; c Warsaw University of Technology, Faculty of Electrical Engineering, Warsaw, Poland; d Center for Medical Image Science and Visualization, Linköping University, Linköping, Sweden

**Keywords:** Triple negative breast cancer, Tumour infiltrating lymphocytes, Artificial intelligence, Multispectral imaging, Prognosis

## Abstract

The tumour microenvironment has been shown to be a valuable source of prognostic information for different cancer types. This holds in particular for triple negative breast cancer (TNBC), a breast cancer subtype for which currently no prognostic biomarkers are established. Although different methods to assess tumour infiltrating lymphocytes (TILs) have been published, it remains unclear which method (marker, region) yields the most optimal prognostic information. In addition, to date, no objective TILs assessment methods are available.

For this proof of concept study, a subset of our previously described TNBC cohort (n = 94) was stained for CD3, CD8 and FOXP3 using multiplex immunohistochemistry and subsequently imaged by a multispectral imaging system. Advanced whole-slide image analysis algorithms, including convolutional neural networks (CNN) were used to register unmixed multispectral images and corresponding H&E sections, to segment the different tissue compartments (tumour, stroma) and to detect all individual positive lymphocytes. Densities of positive lymphocytes were analysed in different regions within the tumour and its neighbouring environment and correlated to relapse free survival (RFS) and overall survival (OS).

We found that for all TILs markers the presence of a high density of positive cells correlated with an improved survival. None of the TILs markers was superior to the others. The results of TILs assessment in the various regions did not show marked differences between each other.

The negative correlation between TILs and survival in our cohort are in line with previous studies. Our results provide directions for optimizing TILs assessment methodology.

## Highlights

• Deep learning algorithms enable objective assessment of biomarkers.
• Automated assessment of tumour infiltrating lymphocytes is feasible.
• Tumour infiltrating lymphocytes are prognostic in triple negative breast cancer.

## 1 Introduction

Breast cancer is the most common type of cancer in women in the world, showing a still increasing incidence. In the Netherlands, an increase in breast cancer incidence from about 8000 women in 1990 to nearly 15,000 in 2015 was observed [1]. To support treatment planning and obtain prognostic information, all invasive breast tumours are routinely classified into histological subtypes according to the WHO classification [2] and assigned a histological grade using the Nottingham grading system [3]. In addition, for prognostic and predictive purposes, all invasive breast tumours are tested for expression of the oestrogen receptor (ER) and progesterone receptor (PR) as well as for overexpression of the human epidermal growth factor receptor 2 (HER2).

Approximately 15% of all breast cancers test negative for these three receptors [4], hence referred to as triple negative breast cancers (TNBC). TNBC has a high incidence among young women [5] and the course of the disease is characterized by a high risk of recurrence in the first three years after initial diagnosis [6]. About one fourth of TNBC patients will develop a recurrence after which the median survival is only 9–13 months [7,8]. Because of the high recurrence risk and the aggressive course of advanced TNBC, early stage TNBC patients will undergo vigorous (loco)regional and systemic treatment. Currently no prognostic biomarkers are available to provide patients and clinicians more personalized guidance. In our previous research, for instance, we have shown that the mitotic density, which is an established prognosticator for breast cancer overall, is not prognostic for this subgroup of patients [9].

The high burden of disease associated with TNBC has been an incentive for extensive research on prognostic and predictive biomarkers. In the last decade, the interplay between host and tumour has gained much attention. The constitution of the microenvironment in which the tumour resides is considered to play a crucial role in the initiation, progression, and invasion of the tumour [10]. Being part of the tumour microenvironment, tumour infiltrating lymphocytes (TILs) have gained particular attention.

TILs are increasingly recognized as a prognostic biomarker in the general breast cancer population [11], as well as in TNBC [12,13]. Even though different studies apply different TIL markers and highly diverse assessment methods, the general conclusion of these studies is that increased TIL density is associated with a better prognosis in TNBC [14]. The presence of specific subsets of lymphocytes is associated with improved survival, particularly for CD8^+^ [15,16] and FOXP3+ [14,17] lymphocytes. However, also an increase of the total amount of mononuclear cells was found beneficial for survival [18]. Attempts to standardize individual evaluation methods for TILs in TNBC are ongoing [19] but do not consider, in a structured manner, which TIL assessment method yields the strongest prognostic information for breast cancer in general or TNBC in particular. To enable clinical validation for this promising biomarker, the most optimal assessment approach in a prognostic context should be determined. This is an important prerequisite to generate maximum patient benefit [20].

The present study aimed to establish the optimal assessment method for immunohistochemically stained TILs in TNBC in relation to patient outcome. To allow accurate identification of the optimal immunohistochemical marker, multiplex immunohistochemistry (mIHC) in combination with spectral image acquisition was used [21]. Tyramide signal amplification (TSA) was used for multiplex immunohistochemistry. Different subsets of TILs (CD8, FOXP3 and CD3 as an overall T-cell marker) in different areas of the tumour and its environment were studied. Advanced image analysis methods based on deep learning were used to generate objective and highly reproducible TIL assessment methods. For this study, we used a subset of a previous established TNBC cohort [8] as discovery cohort.

## 2 Materials and methods

### 2.1 Patients and tissue selections

**Fig. 1:**
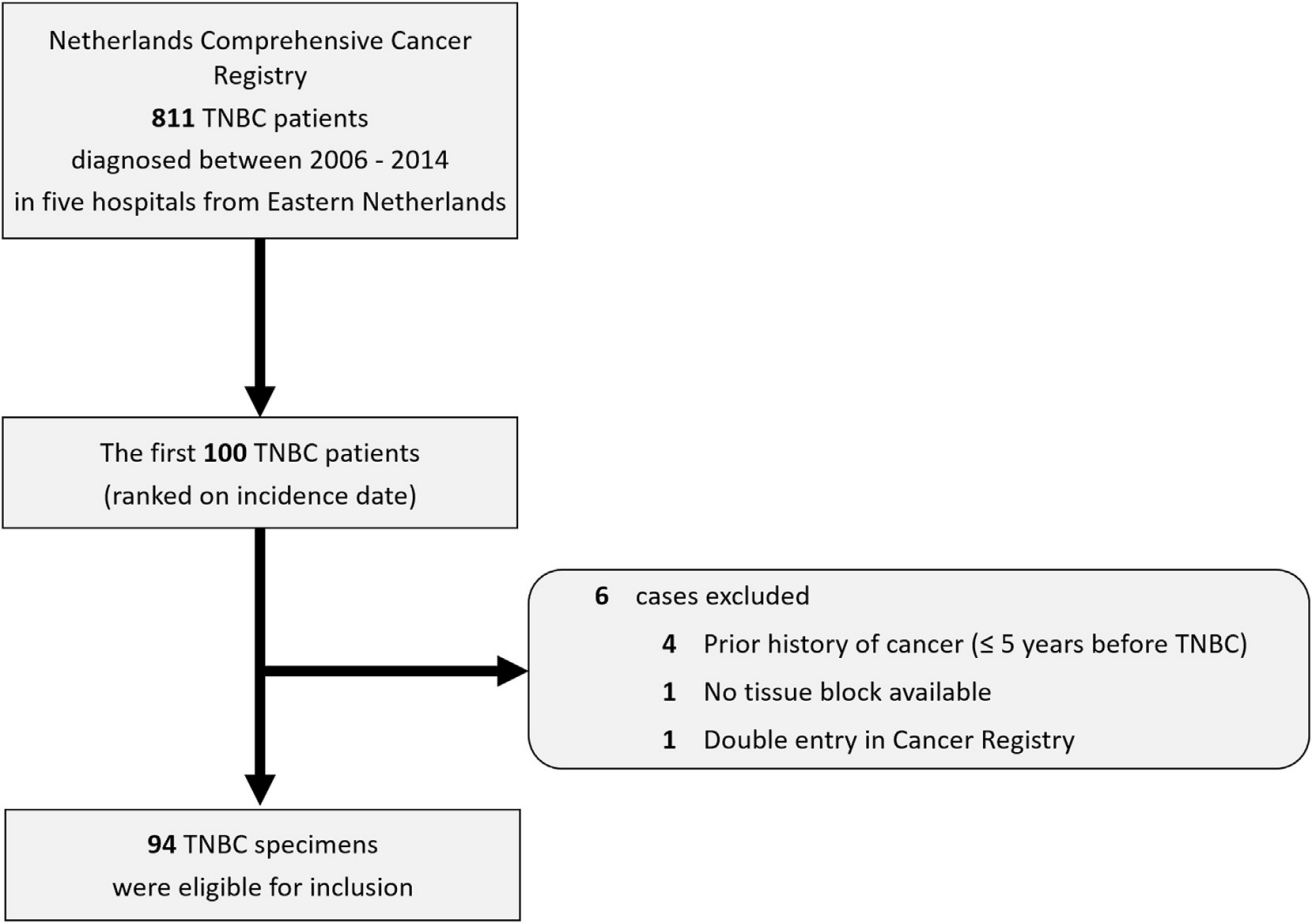
Flow diagram showing the initial number of 811 triple negative breast cancer patients as retrieved from the Dutch Cancer Registry from which 100 patients were selected. During the process of collecting archival tissue blocks and detailed follow up information, 6 patients were excluded from the study. Numbers of excluded patients are listed per factor in descending order.

As part of a previous study, a multicentre, retrospective cohort of stage I-III, non-neoadjuvantly treated TNBC from Eastern Netherlands was assembled using the Netherlands Comprehensive Cancer Registry (IKNL; a nationwide registry in which all malignancies in the Netherlands are registered) [8]. This resulted in a database with a total of 811 patients who were diagnosed between the years 2006 and 2014 in an academic hospital (Radboud University Medical Center (Radboudumc), Nijmegen) or in a general hospital (Canisius Wilhelmina Hospital, Nijmegen; Jeroen Bosch Hospital, ‘s-Hertogenbosch; Bernhoven Hospital, Uden; Hospital Pantein, Boxmeer). Because of the laborious analysis methods used in the present study, a subcohort of 100 patients (Fig. 1) was selected from the previously established TNBC cohort (see further under statistical analysis).

One representative tissue block per tumour was selected based on inspection of archival tissue sections for the presence of invasive cancer with a transition from tumour to normal breast tissue being present [19]. All tumours underwent central histopathological revision for histological subtype and grade (MCAB, PB) according to the prevailing guidelines [2,3]. Both reviewers were blinded for clinicopathological variables and outcome measures (RFS and OS). Clinical and follow up data were retrieved from the Netherlands Comprehensive Cancer Registry (overall survival; OS) and from local patient files (relapse free survival; RFS).

RFS was defined as the time span between the date of diagnosis of TNBC via core needle biopsy/fine needle aspiration and the date of clinically and/or pathologically detected recurrence of TNBC. Hormonal receptor and/or HER2 positive breast cancer occurring after TNBC diagnosis were regarded as a new primary tumour and not as TNBC recurrence. Patients who did not develop a recurrence were censored at the date of last follow up. OS was defined as the interval between date of diagnosis of TNBC and date of death or moment of last follow up. The REMARK guidelines for reporting tumour marker prognostic studies were used [20] and the study was conducted according to the Standards for Reporting of Diagnostic Accuracy (STARD) guideline [22].

### 2.2 Ethical approval

The institutional review board of the Radboudumc waived the requirement for ethical approval (case number 2015–1711). The Dutch codes of conduct for the use of data in health research [23] and for dealing responsibly with human tissue in the context of health research [24] were adhered to.

### 2.3 Multiplex IHC staining procedure

Of every TNBC tissue block, one section was cut to perform the multiplex staining and one for a conventional H&E staining. A panel of six antibodies was composed for the multiplex stainings, from which the results of three antibodies were used in this study (CD3, CD8, FOXP3). The most optimal order and dilutions of antibodies were tested before staining the TNBC cohort.

The most optimal antibody dilutions and order of applying antibodies were tested on formalin fixed paraffin embedded (FFPE) tissue sections of a randomly selected invasive ductal breast cancer from the Radboudumc which was not part of the TNBC cohort. mIHC was optimized by performing a duplex staining consisting of FOXP3 (Opal 620) with CD8 (Opal 650), followed by a triplex staining with the addition of CD3 (Opal 570). All mIHC experiments were performed by repeating staining cycles in series, with microwave treatments in between each cycle and at the end of the mIHC, finished with a DAPI counterstain and enclosed in Fluoromount-G. To confirm optimal dilutions and order of antibodies, FFPE tissue sections of five additional randomly selected invasive ductal breast cancers from the Radboudumc with matching incidence years as the TNBC cohort were used.

### 2.4 Multiplex IHC staining of TNBC cohort

Tissue sections of 3 μm thickness were cut from the TNBC FFPE tumour blocks and subsequently mounted on glass slides. After drying overnight in an oven at 37° Celsius, slides were deparaffinized in xylene, rehydrated and washed in tap water. Epitope retrieval was performed by boiling the slides in citrate buffer (pH 6.0, CBB999; ScyTek) in a microwave. To prevent background staining, protein blocking was performed using TBS-Tween 1% BSA (A7034, Sigma-Aldrich). Primary antibodies FOXP3 (clone 236A/E7; Ebioscience), CD8 (clone C8/144B; DAKO) and CD3 (clone SP7; Thermo Fisher) were incubated for 1 h at room temperature. After several times rinsing in PBS/BSA/Tween, slides were incubated with BrightVision poly-HRP-*anti*-Mouse/Rabbit/Rat IgG (DPVO999HRP; ImmunoLogic) at room temperature for 30 min. The Opal seven-colour IHC Kit (NEL797B001KT, PerkinElmer) which contains fluorophores DAPI, Opal 520, Opal 540, Opal 570, Opal 620, Opal 650 and Opal 690 (NEL703001KT; PerkinElmer) was used to visualize mIHC results. In order to remove the antibody/mIHC complex, a microwave treatment with Tris-EDTA buffer (pH 9) was performed between each staining cycle. Single stain slides were finished with microwave treatment and counterstained with DAPI for 5 min and were enclosed in Fluoromount-G (0100–01; SouthernBiotech). Of every tissue block one extra slide was cut, directly adjacent to the slide used for mIHC, and stained for H&E in the Radboudumc pathology department according to routine practice.

### 2.5 Imaging and image co-registration

**Fig. 2:**
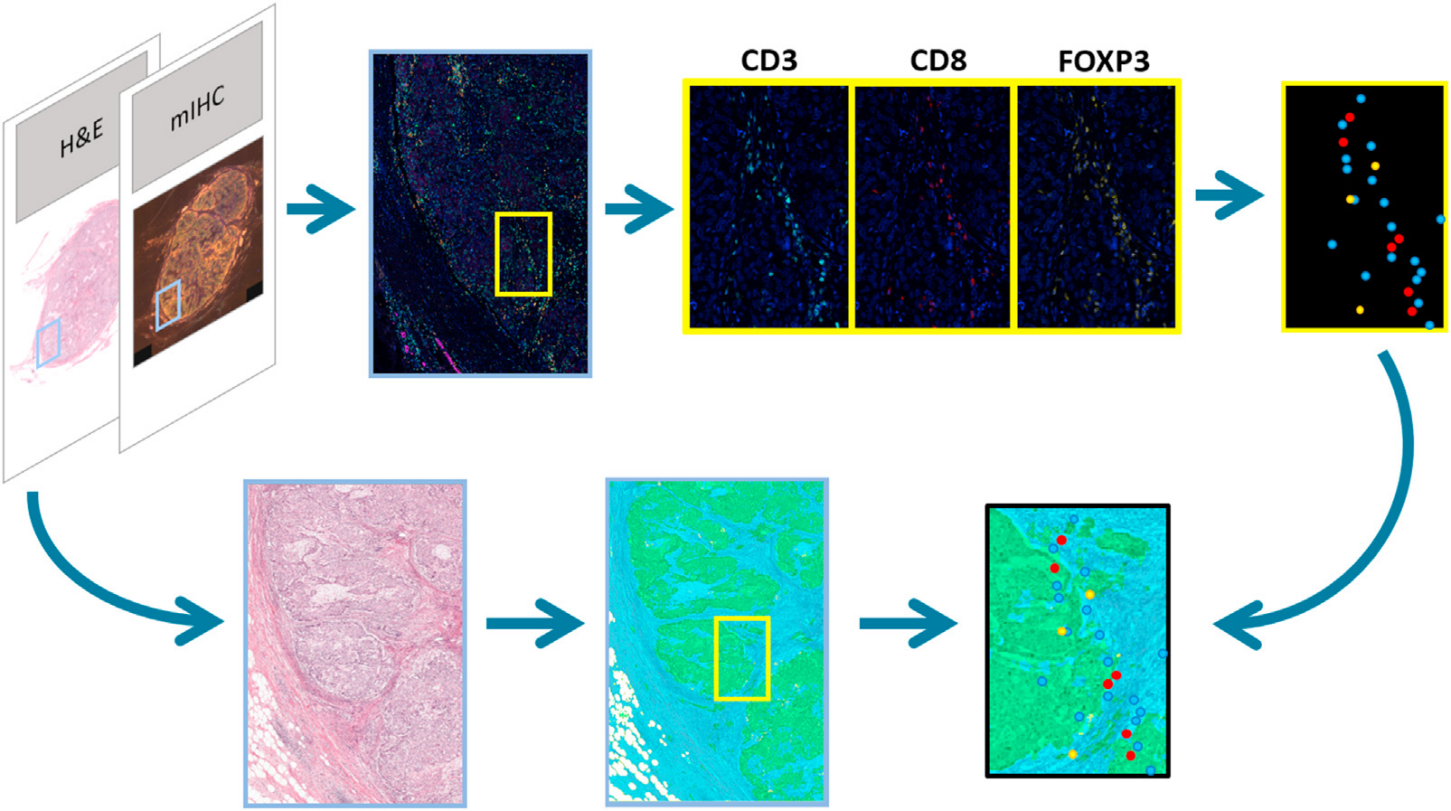
Schematic representation of a part of the TILs assessment work flow. Upper left: for each case a conventional H&E and a mIHC stained section were made and imaged. The different channels in the mIHC image were separated and in the individual images the positive cells were detected for each marker (blue: CD3; red: CD8; yellow: FOXP3). In the H&E image, the epithelial tumour and stroma were segmented for the intratumoural measures (ITA, ITT, ITS). Finally, the mIHC and H&E images were registered which enabled to calculate the density of positive lymphocytes in different areas.

Resulting mIHC slides were imaged using the Vectra spectral imaging system version 2.0.7 (PerkinElmer, Waltham, MA). The Vectra system can acquire mIHC images by recording images at a range of wavelengths, using a spectral camera. Subsequently, the inForm Advanced Image Analysis software (inForm 2.1.1; PerkinElmer) was used to apply spectral unmixing, to reproduce the individual IHC signals in the mIHC staining (Fig. 2, upper part). To be able to perform spectral unmixing, a library of reference signals was built on the basis of each individual single staining of the primary antibodies used in the mIHC and a single staining of DAPI. For background removal, an unstained slide was imaged. Corresponding H&E sections were scanned on a Pannoramic 250 Flash II slide scanner (3DHistech, Hungary) at a spatial resolution of 0.12 μm/pixel. Resulting pairs of H&E and mIHC whole slide images (WSI) were co-registered (i.e. images were aligned such that there is a pixel level correspondence between two images) based on a previous developed algorithm [25,26], which consists of nonlinear registration of whole-slide images***.***

### 2.6 Image analysis

**Fig. 3:**
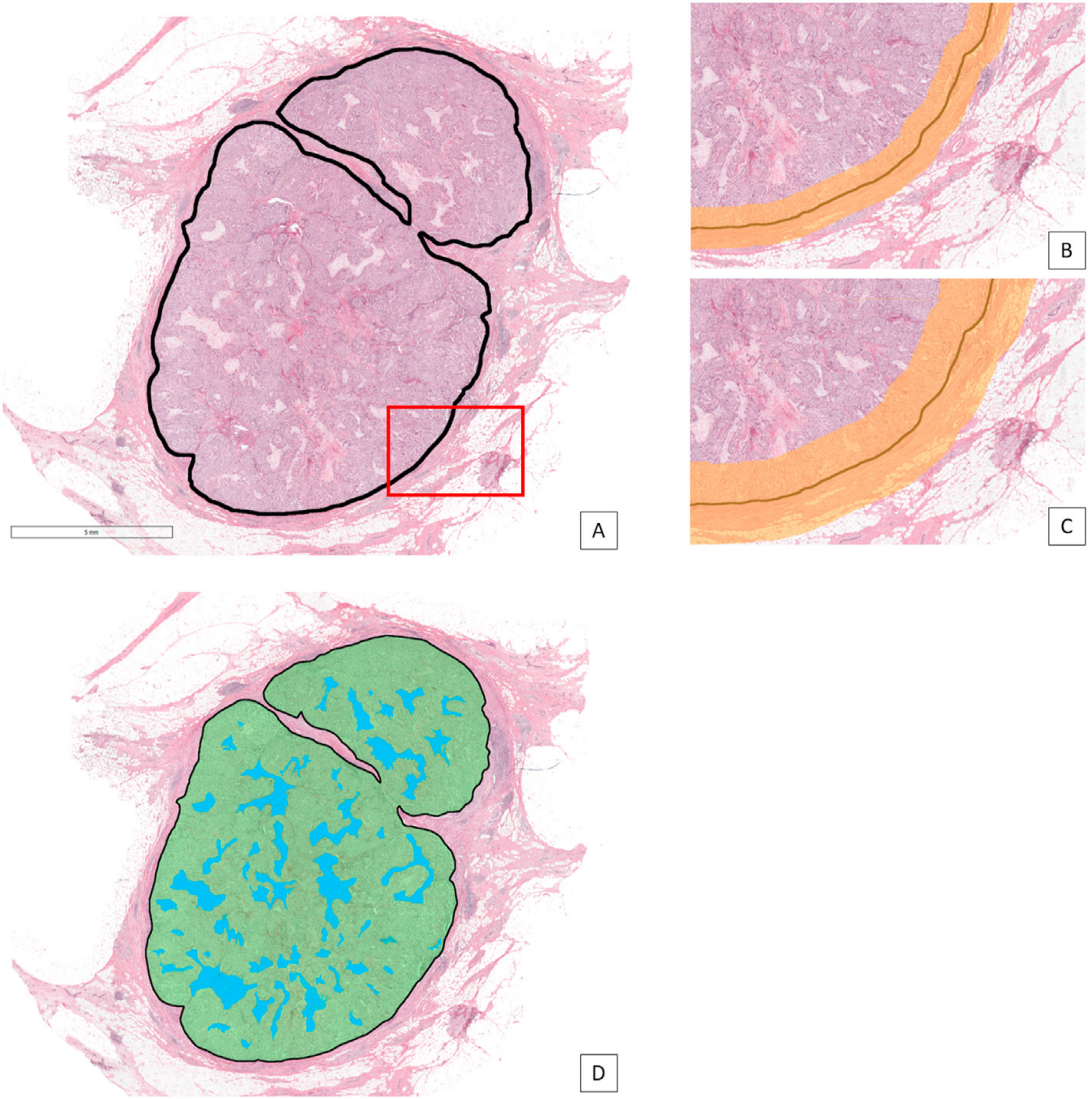
One of the included TNBC cases on which the different regions for TILs assessment are projected. A: Overview of the tumour and adjacent tissue on low magnification. The black line marks the boundary between the epithelial tumour and the adjacent tissue. The red rectangle includes intratumoural and peritumoural area. B: Selected area (red rectangle in A) at high magnification in which an invasive margin of 1 mm is outlined by the orange colour. IM1: density of positive lymphocytes in the total orange area; IM1IO: ratio between the density of positive cells in the inner and outer boundary of the tumoural area. C: Selected area (red rectangle in A) at high magnification in which an invasive margin of 2 mm is outlined by the orange colour. IM2: density of positive lymphocytes in the total orange area; IM2IO: ratio between the density of positive cells in the inner and outer boundary of the tumoural area. D: Identical tumour as in A, in which the intratumoural area was segmented (green: tumour cells; blue: stroma). ITA: density of positive cells in the total intratumoural area (green plus blue area); ITT: density of positive cells in the epithelial tumour regions within the intratumoural area (green area); ITS: density of positive cells in the stromal regions within the intratumoural area (blue area).

TIL densities were assessed in different areas of the tumour and its environment, comparable to methods published previously [19,27,28]. To facilitate such analyses, we used H&E WSIs to identify different components within the invasive tumour (stroma, malignant epithelium) and its neighbouring environment (invasive margin) in which TIL densities were measured. In every digitized H&E slide, the invasive tumour was outlined by an experienced observer (MCAB) (Fig. 3A) who was blinded for clinicopathological variables and outcome measures. To allow for TIL measurements in the invasive margin of the tumours, the annotated tumour bulk outline was dilated on both sides by either 500 μm or 1 mm, resulting in an effective invasive margin of 1 mm and 2 mm, respectively (Fig. 3B and C).

Within the manually outlined tumour bulk area in the H&E section, we applied a previously developed epithelium-stroma segmentation algorithm [29]. This deep learning algorithm was trained on a large number of manually annotated breast cancer cases and is capable of differentiating between regions consisting of epithelial, stromal and fatty tissue. Applying the algorithm resulted in a subdivision of pixels within the tumour bulk as either belonging to the stroma or epithelium class (Fig. 2, lower part, and Fig. 3D). The above described delineation of the tumour and segmentation of the intratumoural area resulted in the following areas in which the TIL density was assessed for CD3, CD8 and FOXP3, as well as ratios between these markers: density of positive cells in the intratumoural area (ITA); density of positive cells in the epithelial tumour regions within the intratumoural area (ITT); density of positive cells in the stromal regions within the intratumoural area (ITS); density of positive cells in a margin of 500 μm on both sides of the boundary of the tumour area (IM1); density of positive cells in a margin of 1 mm on both sides of the boundary of the tumour area (IM2); ratio between the density of positive cells in the 500 μm inner and outer boundary of the tumour area (IM1IO); ratio between the density of positive cells in the 1 mm inner and outer boundary of the tumour area (IM2IO).

A previously developed convolutional neural network to detect individual lymphocytes, positive for the respective markers [30], was applied on the unmixed images (Fig. 2, upper part). Finally, to enable calculation of the density of positive lymphocytes in different areas of the tumour and its neighbouring environment, the detections of positive lymphocytes were projected on the H&E images (Fig. 2, lower right image).

### 2.7 Statistical analysis

For the present study, we selected 100 cases of a previously described cohort as follows [8]. After ranking all patients by incidence date (date of diagnosis with TNBC by either histology or cytology), the first 100 patients were included. To study representativeness of the sub-cohort used in this study against the total TNBC cohort from which cases were taken, the distribution of clinicopathological variables and the of number of events were compared between these two using cross tabulation. No significant differences were observed (p > 0.05; data not shown) using Pearson Chi-Square test. Also, independent samples T tests showed no significant differences (p > 0.05; data not shown) in mean time to events between the cohort used in this study and the total TNBC cohort.

Univariable Cox regression analysis with bootstrapping (5000 bootstraps) was performed for all TIL measures with RFS and OS as primary outcomes. To correct for tumour size, TILs were expressed per 1 mm^2^ area. The number of CD3 and CD8 positive lymphocytes were evaluated per increment of 100 positive cells. Because of the low number of FOXP3 positive cells, this marker was evaluated per increment of 10 positive cells. TIL ratio measures were calculated based on the absolute number of positive cells. All TIL measures were analysed as continuous variables. For all analyses, confidence intervals were set at the 95% level. The baseline alpha level to test for statistical significance was 0.05. This baseline alpha value was not adjusted for multiple comparisons as the aim of this study was comparison of different assessment methodologies without studying the absolute prognostic value of the assessed TIL features. To investigate the prognostic value of TILs in the intratumoural areas more closely, Kaplan Meier curves were produced and Log rank tests were performed. For this, TIL count per increment of 100 positive cells (CD3 and CD8) and per increment of 10 positive cells (FOXP3) per 1 mm^2^ area was dichotomized using the median value of the individual TIL markers as a cut-off. All analyses were performed using statistical software SPSS (version 25.0; IBM, Chicago, USA).

## 3 Results

### 3.1 Patient demographics and tumour characteristics

The Netherlands Comprehensive Cancer Registry provided a cohort of 811 patients who underwent surgery for primary breast cancer between 2006 and 2014 in the 5 participating hospitals from Eastern Netherlands. After ranking patients by incidence date in chronologicalorder, we selected the first one hundred patients for this study. After applying the exclusion criteria and retrieval of archival tissue blocks, 94 tumours remained (Fig. 1) which were stained using mIHC. Table 1 provides an overview of patient and tumour characteristics in the TNBC cohort. The majority of patients were 50 years or older at the time of diagnosis (61.7%). Less than half of the tumours were smaller than 2 cm (43.6%) in size. The prevailing histological subtype was invasive carcinoma of no special type (invasive carcinoma NST) (92.6%). About one in four patients developed a recurrence of TNBC (23.4%) and one in three patients deceased during the follow up period (33.0%). For the patients who were confronted with a recurrence, the median time for developing a clinically detected recurrence was 27.0 months after primary TNBC diagnosis.

**Table 1:** Overview of patient and tumour characteristics of the discovery triple negative breast cancer cohort (n = 94).

	n	%
Gender		
Female	94	100.0
Age, years		
≥50	58	61.7
<50	36	38.3
Primary tumour stage^a^		
T1	41	43.6
T2	47	50.0
T3	6	6.4
T4	0	0
Regional lymph node stage^a^		
N0 (including isolated tumour cells)	51	54.3
N1	27	28.7
N2	7	7.4
N3	5	5.3
Nx (regional lymph nodes cannot be assessed)	4	4.3
Histological grade [3]		
1	0	0
2	14	14.9
3	80	85.1
Histological subtype [2]		
Invasive carcinoma of no special type	87	92.6
Special histological subtypes^b^	7	7.4
Primary surgical treatment		
Mastectomy	37	39.4
Breast conserving surgery	57	60.6
Adjuvant systemic therapy		
None	33	34
Anthracyclines	39	41.5
Anthracyclines with taxanes	20	21.3
Other regimes	3	3.2
Adjuvant radiotherapy		
No	41	43.6
Yes	53	56.4
Development of recurrence^c^		
No	72	76.6
Yes	22	23.4
Deceased (overall)		
No	63	67.0
Yes	31	33.0

a Primary tumour stage and regional lymph node stage were classified according to the TNM 6th edition [31].

b Invasive lobular carcinoma (2 patients), metaplastic carcinoma (1 patient), medullary carcinoma (1 patient), invasive carcinoma with osteoclast like giant cells (1 patient).

c The presence of a recurrence was confirmed either clinically (imaging studies) or with additional pathological examination.

### 3.2 TIL assessment

**Fig. 4:**
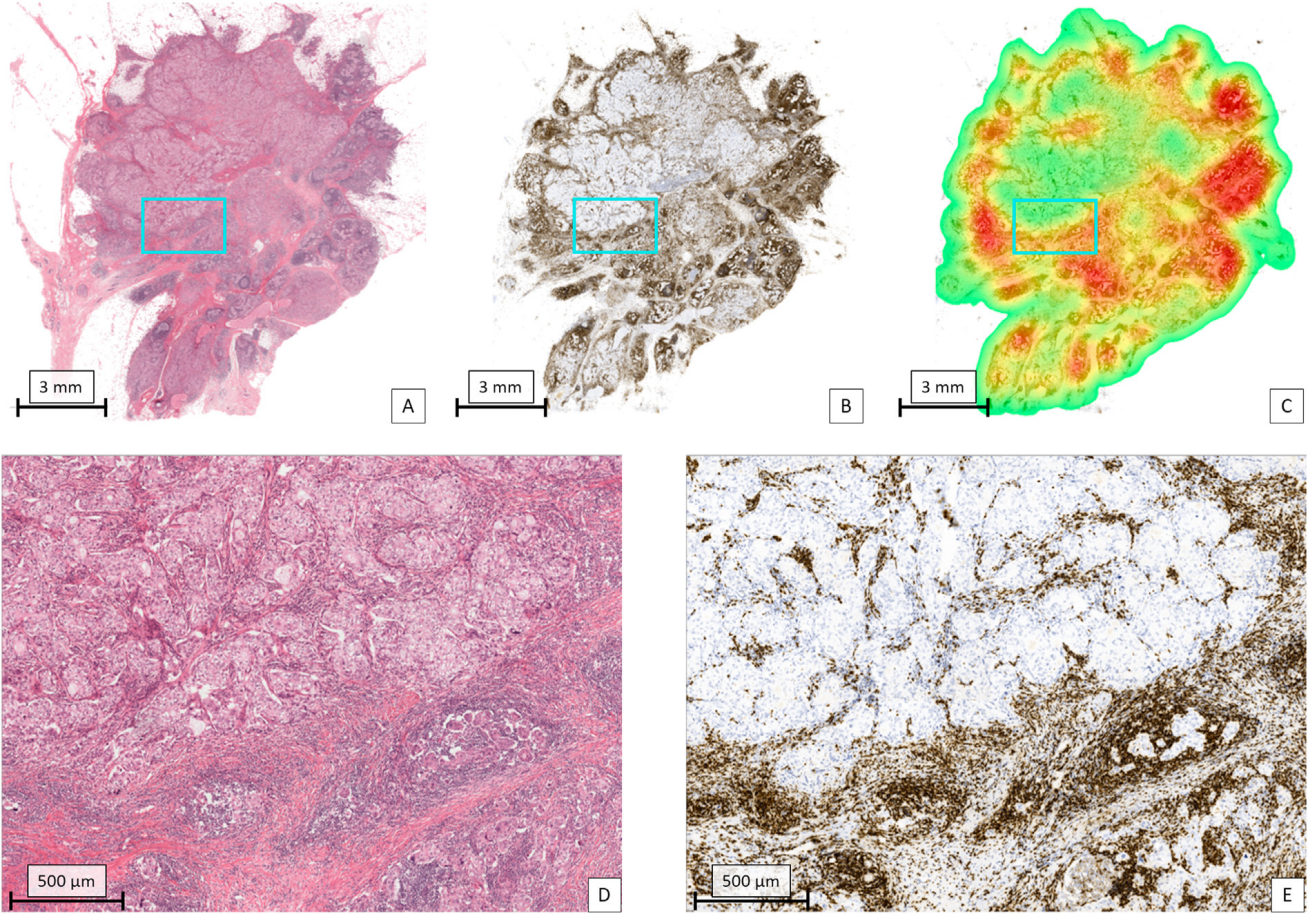
Example of one of the TNBC tumours in the cohort with an abundant lymphocytic infiltrate. A: Overview of the tumour in H&E. The region in the magenta coloured rectangle is shown on a higher magnification in D. B: Result of CD3 staining of the tumour. For visualization purposes, the original immunofluorescent image is displayed in conventional bright field colours. The region in the magenta coloured rectangle is shown on a higher magnification in E. C: Heat map based on the density of CD3 positive lymphocytes with a circle shaped sliding window with an area of 1 mm^2^. Red: high density; yellow: intermediate density; green: low density. D: Selected area (magenta rectangle in A) at high magnification in which an abundant lymphocytic infiltrate is present. E: Selected area (magenta rectangle in B) at high magnification in which a high density of CD3 positive cells is present.

**Fig. 5:**
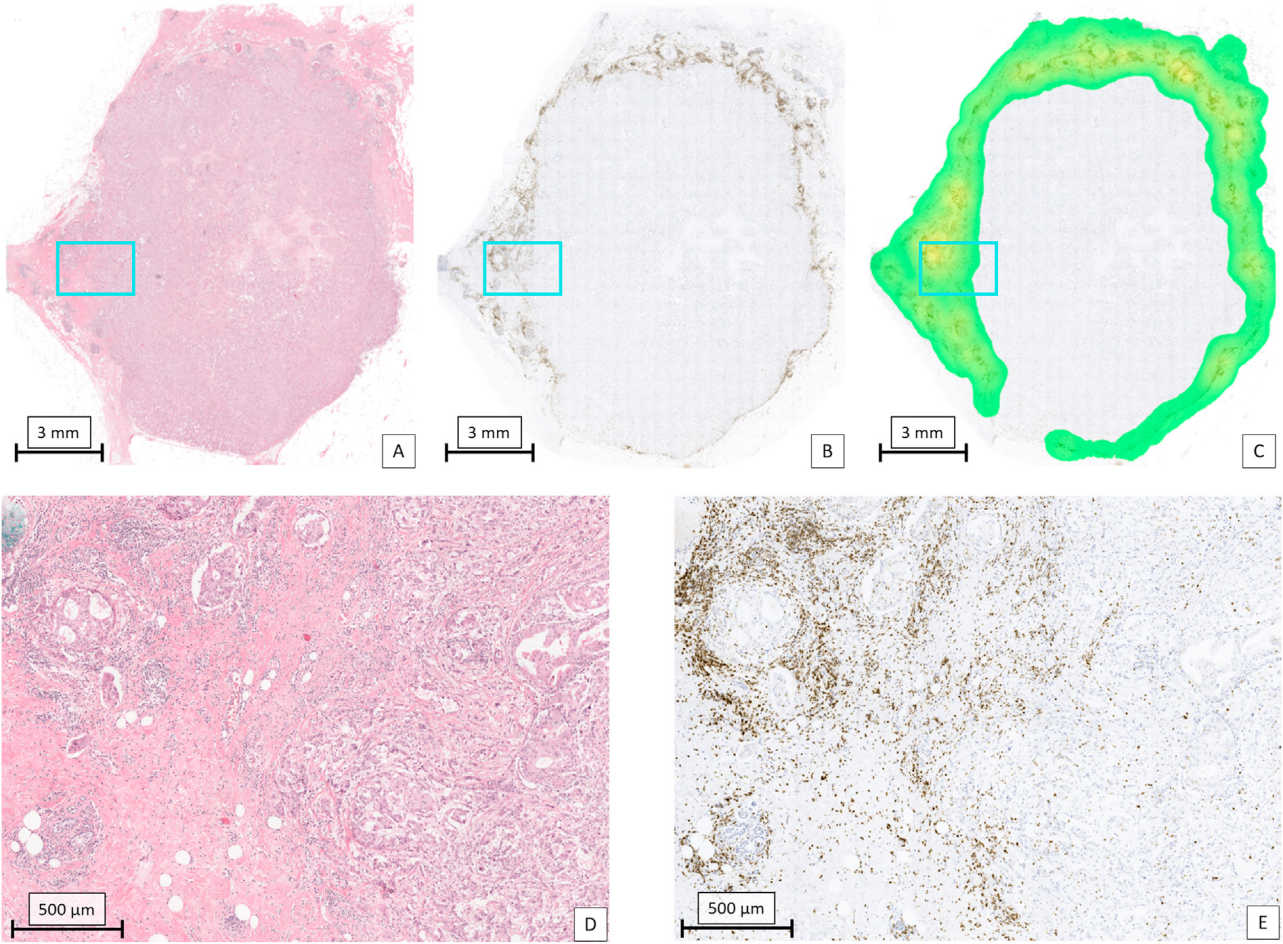
Example of one of the TNBC tumours in the cohort with a sparse lymphocytic infiltrate. A: Overview of the tumour in H&E. The region in the magenta coloured rectangle is shown on a higher magnification in D. B: Result of CD3 staining of the tumour. For visualization purposes, the original immunofluorescent image is displayed in conventional bright field colours. The region in the magenta coloured rectangle is shown on a higher magnification in E. C: Heat map based on the density of CD3 positive lymphocytes with a circle shaped sliding window with an area of 1 mm^2^. Yellow: intermediate density; green: low density. D: Selected area (magenta rectangle in A and B) at high magnification in which a sparse lymphocytic infiltrate is present. E: Selected area (magenta rectangle in B) at high magnification in which a low density of CD3 positive cells is present.

For every patient, the digitized H&E tissue section was co-registered (i.e. images were aligned such that there was a pixel level correspondence between two images) with the digitized mIHC tissue section (Fig. 2). Fig. 3 shows the different regions and measures for the TIL assessment. Different tumours showed a marked different immune response (two examples shown in Fig. 4, Fig. 5).

### 3.3 Effect of individual TIL markers

Table 2 shows that the presence of CD3, CD8 and FOXP3 all correlated with an improved survival. CD3 seemed to be slightly more prognostic than FOXP3 and CD8, in terms of lower p-values, with CD8 being least favourable. Generally, correlation between TILs and relapse free survival was weaker than with overall survival. In contrast to the use of individual markers, the ratios between markers generally correlated poorly with prognosis. Supplementary table 1 shows p-values of the Log rank tests of Kaplan Meier curves when analysing TIL markers in the intratumoural areas (ITA, ITT, ITS) as a dichotomous variable using the median as a cut-off. For both RFS and OS, a binary classification of CD3 and CD8 counts yielded significant differences in survival between high and low TIL counts. In these analyses, a high TIL count correlated with improved survival.

**Table 2:** Univariable analyses for relapse free survival and overall survival for the triple negative breast cancer cohort (5000 bootstraps).

	Relapse free survival HR (95% CI)	p value	Overall survival HR (95% CI)	p value
CD3				
ITA^a^	0.899 (0.771–0.974)	0.065	0.890 (0.782–0.959)	0.019^c^
ITT^a^	0.888 (0.721–0.969)	0.089	0.883 (0.758–0.956)	**0.032**^c^
ITS^a^	0.915 (0.822–0.978)	**0.046**^c^	0.908 (0.829–0.964)	**0.011**^c^
IM1^a^	0.914 (0.847–0.969)	**0.008**^c^	0.901 (0.833–0.954)	**0.002**^c^
IM2^a^	0.888 (0.803–0.955)	**0.009**^c^	0.878 (0.787–0.946)	**0.004**^c^
IM1IO	0.461 (0.150–0.921)	0.081	0.987 (0.423–1.153)	0.969
IM2IO	0.597 (0.254–0.984)	0.134	1.001 (0.555–1.408)	0.995
CD8				
ITA^a^	0.826 (0.457–0.969)	0.204	0.791 (0.515–0.922)	0.073
ITT^a^	0.824 (0.345–0.975)	0.257	0.788 (0.464–0.926)	0.115
ITS^a^	0.826 (0.546–0.969)	0.131	0.805 (0.592–0.930)	**0.041**^c^
IM1^a^	0.826 (0.651–0.942)	**0.036**^c^	0.801 (0.637–0.913)	**0.013**^c^
IM2^a^	0.777 (0.551–0.919)	**0.045**^c^	0.756 (0.557–0.894)	**0.014**^c^
IM1IO	0.508 (0.188–0.975)	0.113	0.836 (0.356–1.344)	0.596
IM2IO	0.654 (0.286–1.234)	0.181	0.854 (0.483–1.198)	0.491
FOXP3				
ITA^b^	0.694 (0.416–0.837)	**0.020**^c^	0.728 (0.545–0.845)	**0.005**^c^
ITT^b^	0.705 (0.396–0.598)	0.050	0.722 (0.505–0.853)	**0.013**^c^
ITS^b^	0.749 (0.517–0.868)	**0.019**^c^	0.781 (0.624–0.875)	**0.003**^c^
IM1^b^	0.846 (0.640–0.945)	0.083	0.901 (0.694–0.982)	0.157
IM2^b^	0.794 (0.549–0.909)	0.061	0.860 (0.631–0.962)	0.095
IM1IO	0.457 (0.196–0.753)	**0.031**^c^	0.997 (0.455–1.176)	0.989
IM2IO	0.625 (0.356–0.850)	**0.032**^c^	1.005 (0.645–1.121)	0.960
CD8/CD3 ratio				
ITA	0.000 (0.00 – ∞)	NA	0.000 (0.00 – ∞)	NA
ITT	0.000 (0.00 – ∞)	NA	0.000 (0.00 – ∞)	NA
ITS	0.000 (0.00 – ∞)	NA	0.000 (0.00 – ∞)	NA
IM1	0.000 (0.00 – ∞)	NA	0.000 (0.00 – ∞)	NA
IM2	0.000 (0.00 – ∞)	NA	0.000 (0.00 – ∞)	NA
IM1IO	0.451 (0.077–1.844)	0.292	0.242 (0.045–0.815)	**0.049**^c^
IM2IO	0.551 (0.142–1.706)	0.338	0.264 (0.064–0.825)	**0.037**^c^
FOXP3/CD3 ratio				
ITA	0.000 (0.00 – ∞)	NA	0.000 (0.00 – ∞)	NA
ITT	0.000 (0.00 – ∞)	NA	0.000 (0.00 – ∞)	NA
ITS	0.000 (0.00 – ∞)	NA	0.000 (0.00 – ∞)	NA
IM1	0.000 (0.00 – ∞)	NA	0.000 (0.00 – ∞)	NA
IM2	0.000 (0.00 – ∞)	NA	0.000 (0.00 – ∞)	NA
IM1IO	0.780 (0.281–1.528)	0.491	0.881 (0.454–1.752)	0.575
IM2IO	0.857 (0.441–1.449)	0.518	0.935 (0.575–1.770)	0.681
CD8/FOXP3 ratio				
ITA	0.000 (0.00 – ∞)	NA	0.000 (0.00 – ∞)	NA
ITT	0.000 (0.00 – ∞)	NA	0.000 (0.00 – ∞)	NA
ITS	0.000 (0.00 – ∞)	NA	0.000 (0.00 – ∞)	NA
IM1	0.000 (0.00 – ∞)	NA	0.000 (0.00 – ∞)	NA
IM2	0.000 (0.00 – ∞)	NA	0.000 (0.00 – ∞)	NA
IM1IO	0.982 (0.362–1.781)	0.956	1.109 (0.640–2.032)	0.662
IM2IO	0.925 (0.500–1.384)	0.736	1.064 (0.722–1.714)	0.703

Abbreviations.

CI: confidence interval.

HR: hazard ratio.

NA: not applicable; NST: no special type.

ITA: Density of positive cells in the intratumoural area.

ITT: Density of positive cells in the epithelial tumour regions within the intratumoural area.

ITS: Density of positive cells in the stromal regions within the intratumoural area.

IM1: Density of positive cells in a margin of 500 μm on both sides of the boundary of the tumour area.

IM2: Density of positive cells in a margin of 1 mm on both sides of the boundary of the tumour area.

IM1IO: Ratio between the density of positive cells in the 500 μm inner and outer boundary of the tumour area.

IM2IO: Ratio between the density of positive cells in the 1 mm inner and outer boundary of the tumour area.

a per increment of 100 positive lymphocytes.

b per increment of 10 positive lymphocytes.

c No longer significant after correction for multiple comparisons for alpha = 5%.

### 3.4 Effect of region of TIL assessment

Assessment of TILs intratumourally or in the invasive margin showed comparable correlation with survival, with some variations between the different markers: higher TIL densities in these areas were associated with a survival benefit (RFS HR varying between 0.777 (CD8, IM2) and 0.915 (CD3, ITS); OS HR varying between 0.722 (FOXP3, ITT) and 0.908 (CD3, ITA)). We did not observe clear differences between intratumoural measurement overall versus in tumour nests only or stroma only: for instance the HR for RFS for CD3 overall in the tumour is 0.899 while limiting the analysis to either tumour (HR is 0.888) or stroma (HR is 0.915) did not markedly change the HR. The same phenomenon is seen for CD8 and FOXP3 and the OS. Also, the size of the invasive margin in which TIL density was assessed (1 mm vs 2 mm) did not affect the prognostic value. In addition, the ratio between the inner and outer margin of the invasive margin (IM1IO, IM2IO) did not yield any relation with survival, except from FOXP3 for RFS.

## 4 Discussion

In this study, we explored different methods to objectively assess TILs in immunohistochemically stained sections of TNBC and to relate this to patient outcome. We studied three TIL markers (CD3, CD8 and FOXP3) in various regions within the tumour and in its adjacent environment. To objectively assess TILs, we used automated analysis based on deep learning, which can detect each individual positive lymphocyte. Our results showed that in general the abundance of TILs was negatively correlated with RFS and OS, with minor differences between used markers or definition of measurement region (e.g. intratumourally, tumour periphery, etc.). Using ratios between markers (e.g. the CD3/CD8 ratio) was found to be poorly prognostic and should therefore be avoided.

Characterization of the tumour associated immune infiltrate in breast cancer has gained widespread attention of the scientific community in the last decade. Many studies concluded that there is prognostic value of TILs for breast cancer. A uniform and well-established assessment method, however, is currently still lacking and a variety of methods to assess TILs have been published [19,27,28,34]. As a result, published studies are difficult to compare and guidance for larger, prospective validation studies is lacking. Typically, protocols for visual TIL assessment take into account feasibility (should be executable within a limited amount of time and budget, by a sufficiently trained human) and are often based on understanding of (hypothesized) underlying pathological processes. For instance, the International TIL Working Group (ITWG) has published several guidelines to assess TILs in breast cancer [19,32]. Their proposed method consists of visually estimating the percentage of mononuclear inflammatory cells in the intratumoural stromal area in an H&E section. The reason behind this is that using only H&E reduces costs (compared to more specific TIL markers applying IHC), but makes it difficult for humans to recognize TIL’s within tumour nests [32,33]. Therefore, only TILs in tumour stroma are counted. In particular for HER2 positive and TNBC, they correlated a higher stromal TIL presence to an improved survival [18,33], which, combined with the ease of use, makes it a strong candidate for inclusion in guidelines. However, a data-driven approach in which the optimal protocol for biomarker assessment against the desired outcome (e.g. overall survival) is studied might lead to stronger biomarkers. In the present study, computational pathology techniques were instrumental in enabling such analysis, as it is not possible to assess a larger number of quantitative descriptors in a large series of cases.

In the present study we focused on TNBC, applying the most common TIL markers using measuring approaches as published by different research groups [19,27,28]. We could, for instance, show that only including the tumour stroma and ignoring TILs within tumour nests, as prescribed by the ITWG, does not reduce prognostic value as compared to counting TILs within the entire tumour region. The finding that the choice of marker or measurement region has minor effect on prognostic value implies that in general TILs are a robust and reliable biomarker for this group of patients, as the marker is not overly sensitive to methodological variables.

Although the main focus of the Immunoscore consortium is colon cancer, the methodology of this research group has been of great influence in the field of TILs in cancer [27]. The Immunoscore method consists of assessment of CD3 and CD8 densities both in the tumour and invasive margin regions (yielding 4 scores). These are subsequently translated into percentiles and averaged, after which the average is translated into a so-called ‘immunoscore’ (high, intermediate and low). It is not straightforward to compare the extensive immunoscore protocol with results from the present study. We have observed that both intra-tumoural TILs and TILs at the tumour margin contain prognostic information. We have also found that combining multiple markers (by calculating ratios) does not yield prognostic information, making it questionable whether more than one single marker is needed for TIL assessment in TNBC.

In melanoma research, the ratio between the intratumoural and peritumoural T cell density (I/P ratio) was shown to correlate with the survival [28] for patients who had been diagnosed with distant metastases of their melanoma. Patients with a higher I/P ratio had a longer survival. In the present study, the intratumoural to peritumoural ratio in our TNBC cohort (IM1IO, IM2IO) was generally not prognostic. This may be attributed to different immune-regulation mechanisms for different types of malignancies.

In the present study we used multiplex IHC and deep learning, to prevent being hampered by the subjectivity of the human eye. Even though it may be felt that the use of IHC, whole slide imaging and machine learning will only be available in a limited number of diagnostic settings, we believe that the general trend of digitization of pathology diagnostics will result in wide-scale implementation of AI for histopathology. The results from the present study can be translated into essays based on straightforward single IHC staining protocols using DAB, making them available for any sufficiently equipped pathology laboratory.

Our study has several strengths. The patients within this study were selected from a multicentre TNBC cohort which consists of patients from 5 different hospitals, including both academic and general hospitals. To objectify TIL assessment, we used previously developed machine learning algorithms, preventing human intra- and interobserver variability. To optimize comparison between markers, we used mIHC combined with spectral imaging. This study is limited by the constraints of a retrospective analysis. However, a considerable effort was made to obtain high quality and complete follow up data. The extensive analysis with mIHC made it infeasible to study more than the currently included 100 patients.

We used an existing deep learning algorithm for automated delineation of regions containing epithelium, which was trained on common adenocarcinomas. Because TNBC tumours display a wider variation in morphological appearance as compared to general breast cancer, the delineation of tumour cells was unsatisfying for some tumours. As detailed manual outlining of individual tumour cells is not feasible, we had to take these segmentation results for granted, adding some noise to the ITT and ITS measures.

In conclusion, we studied the prognostic values of TILs in TNBC using completely automated assessment methods and IHC. The suggested prognostic value of TILs in our study are in line with previous research, and provide directions for optimizing TILs assessment methodology. This paper proposes a structured framework for optimizing automated TILs assessment which is preferred to application of a single (potentially suboptimal) method. Larger studies are needed to find out if one of the used image analysis algorithms is superior in predicting survival.

## Funding

This study was funded by a Junior Researcher grant from the 10.13039/501100006209Radboud University Medical Center Institute for Health Sciences (10.13039/501100017246RIHS).

## Declaration of competing interest

Jeroen van der Laak is member of the scientific advisory boards of Philips, the Netherlands and ContextVision, Sweden and receives research funding from Philips, the Netherlands and Sectra, Sweden. Geert Litjens received research funding from Philips Digital Pathology Solutions (Best, the Netherlands) and has a consultancy role for Novartis (Basel, Switzerland). The other authors have no conflicts of interest to disclose.
