# Supplementary material for: Optimized tumour infiltrating lymphocyte assessment for triple negative breast cancer prognostics
Source: Breast. 2021 Feb 17;56:78–87. doi: 10.1016/j.breast.2021.02.007 (PMC7933536; doi:10.1016/j.breast.2021.02.007)
Supplement: Multimedia component 1 [file mmc1.docx]

Supplementary table 1: P-values of log rank tests for the comparison of binary survival curves of TILs using the median as a cut-off.

|  | Relapse free survival  p-value | Overall survival  p-value |
| --- | --- | --- |
| CD3 | | |
| ITA* | 0.006 | 0.003 |
| ITT* | 0.001 | 0.001 |
| ITS* | 0.022 | 0.010 |
|  | | |
| CD8 | | |
| ITA* | 0.001 | 0.001 |
| ITT* | < 0.001 | < 0.001 |
| ITS* | 0.023 | 0.011 |
|  | | |
| FOXP3 | | |
| ITA** | 0.404 | 0.226 |
| ITT** | 0.404 | 0.226 |
| ITS** | 0.343 | 0.177 |
|  | | |
| * per increment of 100 positive lymphocytes  ** per increment of 10 positive lymphocytes  Abbreviations:  ITA: Density of positive cells in the intratumoural area;  ITT: Density of positive cells in the epithelial tumour regions within the intratumoural area  ITS: Density of positive cells in the stromal regions within the intratumoural area | | |
